# Reviews

**Published:** 1881-07

**Authors:** 


### Reviews.

---

**Transactions of the Medical Society of the State of New York for the Year 1880.**

It is not strange that a creditable collection of papers should be furnished by the representative Medical Society of a large state like New York. There are a few in this volume of the

Transactions, which can hardly be considered up to the average, but most contain new facts, or familiar ones presented in a new light. It is evidently impossible to consider these in detail, or even to single out some one or two for special mention. But wherever we find articles from the pens of Doctors Gray, Moore, Sims, Post, Buckley, Wier, and others of equal eminence, we may be certain there are also many pages well worth perusal.

---

**A Treatise on the Diseases of the Nervous System.** By WILLIAM A. HAMMOND, M. D. Seventh edition. Re-written, enlarged and improved. New York: D. Appleton & Co.

The author needs no introduction to the medical public, having been a conspicuous figure in this department of enquiry for several years. Since the first appearance of the work, in 1871, its merits have been universally recognized, and it has been carefully perused by many a practitioner, who sought for light upon some obscure form of "nervous" trouble. More than once too, when questions have arisen as to the existence, or nature of suspected insanity, this work has been called into requisition, and the clear statements have usually pointed out the way to a solution of the problems. Unfortunately there is much—very much—yet to be learned, concerning diseases of the nervous system, but the improvements in this edition over the last, show that workers in that department are not idle. Concerning one point, however, we must take issue with the Doctor; for, when he would lay such special stress upon the aid of the ophthalmoscope in diagnosing cerebral congestion or allied troubles, we are forced to regret that this instrument—invaluable as it is for other purposes—can not be relied upon so implicitly, and oculists seem to agree quite generally, that the condition of the retina and optic disk vary too much within healthy limits to warrant the conclusions drawn by Dr. Hammond. Otherwise it is a most excellent work and can only be mentioned in terms of commendation.

